# BDNF, proBDNF and IGF-1 serum levels in naïve and medicated subjects with autism

**DOI:** 10.1038/s41598-022-17503-6

**Published:** 2022-08-12

**Authors:** Maria de los Angeles Robinson-Agramonte, Bernadeta Michalski, Belkis Vidal-Martinez, Leyanis Ramos Hernández, Mabel Whilby Santiesteban, Margaret Fahnestock

**Affiliations:** 1 Neuroimmunology Department, International Center for Neurological Restoration, Havana, Cuba; 2 Department of Psychiatry and Behavioural Neurosciences, McMaster University, 1280 Main St. West, Hamilton, ON L8S 4K1 Canada; 3 Children’s Hospital Borrás-Marfán, Havana, Cuba; 4 Psychiatry Department, Children’s Hospital Las Católicas, Havana, Cuba

**Keywords:** Neuroscience, Diseases, Molecular medicine

## Abstract

Brain-derived neurotrophic factor (BDNF) and insulin-like growth factor 1 (IGF-1) promote the development and maintenance of neural circuits. Alterations in these factors might contribute to autism spectrum disorder (ASD). We asked whether serum BDNF, proBDNF, and IGF-1 levels are altered in an ASD population compared to controls. We measured serum BDNF, proBDNF, and IGF-1 immunoreactive protein in boys and girls aged 5–15 years old with mild to moderate ASD and non-autistic controls by ELISA. IGF-1 was increased in ASD serum compared to controls and was correlated with age and with CARS scores. Serum BDNF levels did not differ between groups, however, proBDNF serum levels were decreased in subjects with ASD compared to non-autistic controls. Medicated, but not unmedicated, ASD subjects exhibited lower serum proBDNF levels compared to controls, while neither IGF-1 nor BDNF levels differed between treatment groups. These data support the involvement of proBDNF and IGF-1 in the pathogenesis and treatment of autism.

## Introduction

Autism spectrum disorder (ASD) is defined by persistent deficits in communication and social interaction accompanied by restricted, repetitive patterns of behavior, interests, or activities^1^. In addition to core symptoms, many individuals with ASD have co-occurring medical conditions, most frequently developmental or psychiatric conditions. The worldwide prevalence of ASD is approximately 1%, and more males than females are affected, although evidence suggests that autism in females may be under-diagnosed^2^. While synaptic dysfunction leading to impaired establishment and maintenance of functional neuronal networks occurs in ASD, the molecular mechanisms remain unclear^3,4^. Studies in humans with ASD and corresponding animal models have demonstrated that alterations of neurotrophic factor levels and their associated signaling pathways might contribute to the underlying disease pathophysiology^5–9^.

Neurotrophic factors are secreted proteins promoting the development, maintenance and function of neural circuits. They are essential regulators of neuronal maturation including synaptogenesis. The role of brain-derived neurotrophic factor (BDNF) and insulin-like growth factor 1 (IGF-1) has been investigated in ASD. Altered BDNF signaling is implicated in ASD pathology, while IGF-1 is a potential candidate for treatment in this disorder^8,10,11^.

BDNF plays a pivotal role in the development and plasticity of the brain and is particularly important for the development of neural circuits and the regulation of glutamatergic and GABAergic synapses^12–14^. BDNF contributes to pre- and postnatal brain development and survival, has potent effects on synaptic and structural plasticity, strengthens excitatory (glutamatergic) synapses and weakens inhibitory (GABAergic) synapses^15,16^. BDNF binds to its receptor (TrkB) to activate canonical survival and neurite outgrowth signaling pathways such as the mitogen-activated protein kinase (MAPK) and phosphatidylinositol-3 kinase (PI3K) serine-threonine-specific protein kinase (AKT1) pathways^17,18^, while its precursor, proBDNF, binds to the pan-neurotrophin receptor (p75NTR) to signal neurite retraction and apoptosis^19–21^. BDNF immunoreactivity measured by ELISA detects both mature BDNF and proBDNF, whereas proBDNF-specific ELISA or Western blotting distinguishes the isoforms. Higher levels of BDNF immunoreactivity have been found in post-mortem brain tissue of subjects with autism^9,22^, at least in part due to elevated proBDNF^9^. Elevated proBDNF may increase signaling through the p75NTR-RhoA pathway, which opposes the Rac pathway, and may contribute to aberrant connectivity in ASD^5^. In addition, TrkB receptor and signaling through the PI3K-AKT1 pathway are reduced in idiopathic autism compared to controls^8^. ProBDNF levels have not been measured in peripheral blood of subjects with autism, whereas BDNF immunoreactivity measured by ELISA in peripheral blood of children with autism compared to controls has resulted in mixed findings. However, systematic reviews and meta-analyses found increased BDNF blood levels (serum and plasma) in subjects with autism^23–25^.

The members of the insulin-like growth factor gene family, insulin-like growth factors 1 and 2 (IGF-1, IGF-2), play a key trophic role in the central nervous system (CNS). IGF-1 acts on its receptor (IGF1R) to activate the same signaling pathways as BDNF, the MAPK and PI3K-AKT1 pathways^26,27^, which play a crucial role in synaptic protein expression and inhibition of apoptosis. IGF-1 improves aberrant brain network connectivity, which is known to be an important neurobiological feature in ASD^6,11^, possibly by normalizing PI3K-AKT1 signaling. IGF-1 levels have been measured in brain parenchyma, CSF, plasma, serum and urine in autistic compared to control subjects, and the results are mixed^28–34^. CSF IGF-1 is significantly lower only in autistic children under 5 years old, whereas there is no IGF-1 difference in CSF or most brain areas in older children^29,30,33^. In contrast, serum and plasma levels of IGF-1 are reportedly higher in children with autism than controls^32,34^.

The development of effective pharmacological treatments for human psychiatric and neurological disorders, including ASD, represents one of the most challenging fields of neuroscience, due mainly to current limitations in our understanding of neurodevelopment, neurological function, and their links to psychiatry. Pharmacological treatment of ASD is focused on symptoms and not on progression of the disorder. Treatment is complicated by the heterogeneity of ASD and the presence of comorbid psychiatric illnesses or conditions in the majority of cases^35^. Blood BDNF levels have been highlighted as a potential biomarker of therapeutic efficacy^36^. Indeed, some of the drugs used to treat ASD and its accompanying comorbidities, such as depression, are known to affect BDNF levels^37^. However, there is still a gap in our understanding of the mechanisms underlying the differential changes in BDNF levels in the periphery and brain and their impact following pharmacological interventions in this disease.

The current study aimed to compare BDNF, proBDNF and IGF-1 serum levels between children aged 5–15 years old with mild to moderate autism and controls. Because pharmacological treatments can influence serum levels of these molecules, in addition to considering the groups as a whole, in this study we also separately analyzed BDNF, proBDNF and IGF-1 levels and the ratio of proBDNF/total BDNF in medication-naïve subjects versus those on medication. There are many gaps in our understanding of autism etiology, which limits its clinical management and treatment. These neurotrophic factors may not only provide blood biomarkers but may also offer clues to the pathophysiology of the disorder.

## Materials and methods

### Study subjects

The analysis included 22 children with a clinical diagnosis of ASD from the ambulatory services of the International Center for Neurological Restoration (CIREN), Borrás-Marfán Hospital and Las Católicas Hospital (Havana, Cuba) and 29 typically developing controls at similar ages. The control group included typically developing children gathered from the Surgery Service of both hospitals, which required blood for routine analysis prior to surgery. The age range of the subjects included in this study was 5–15 years old (Tables 1 and 2). The mean age of the control group was 8.68 ± 0.54 years and 9.45 ± 0.63 years for the autistic group. A 2-tailed unpaired *t*-test confirmed that there was no difference in age between the groups (p = 0.396). The control group contained 8 females and 21 males, the autistic group 5 females and 17 males (Tables 1 and 2). On average, 25% of the analyzed serum samples were from female subjects.

**Table 1:** Characteristics of subjects with autism.

ID#	Age years	Sex	BDNF ng/ml	ProBDNF ng/ml	ProBDNF/total BDNF	IGF-1 ng/ml	T/H	CGI-S	ABC-T	ADI-T	ATEC-T	CARS
A1	5	M	40.58	10.03	0.247	115.04	CBZ	4	99	44^a^	70	28.5
A16	5	F	29.11	11.35	0.390	113.36	CBZ	4	87	74	38	36
A17	6	M	48.65	28.45^a^	0.585	76.09	No Tx	3	98	63	46	26
A2	7	M	39.97	10.52	0.263	98.19	CBZ	3	81	69	36	26
A3	7	M	40.67	10.29	0.253	314.73^c^	No Tx	4	89	65	36	29.5
A18	7	F	19.71	10.38	0.526	93.01	VPA	4	121	69	62	33.5
A19	7	M	43.7	10.36	0.237	114.21	No Tx	4	85	72	57	28
A4	8	F	42.46	9.9	0.233	288.34	CBZ	4	121	64	70	32
A5	8	M	30.49	9.93	0.326	72.46	CBZ	3	102	61	46	26
A6	8	F	36.29	21.08	0.581	96.92	No Tx	3	110	70	58	27.5
A7	9	M	29.56	18.79^b^	0.636	134.91	CBZ	2	89	69	41	24
A8	9	M	33.94	9.66	0.285	125.33	CBZ	3	103	65	20	29
A9	10	M	35.85	6.01	0.168	105.47	CBZ	4	85	74	59	31.5
A10	10	F	26.79	14.43	0.538	104.27	MTPHE	3	94	65	62	28.5
A11	11	M	36.86	11.38	0.309	322.93	HLP	3	85	64	44	36
A20	11	M	39.23	15.95	0.407	92.4	No Tx	3	89	63	41	27.5
A12	13	M	42.3	7.5	0.177	228.1	CBZ	3	108	58	44	29.5
A13	13	M	40.64	11.85	0.292	255.01	CBZ	4	82	74	36	36.5
A14	13	M	28.79	7.77	0.27	143.17	CBZ + R	4	161^a^	69	103^a^	35
A15	13	M	36.67	9.31	0.254	177.35	No Tx	4	95	58	39	35
A21	13	M	35.95	9.4	0.262	164.83	No Tx	2	81	67	37	24
A22	15	M	43.83	19.31	0.441	131.78	No Tx	4	102	62	45	30
Mean	9.45	M = 17	36.46	12.44	0.349	153.09						
SEM ±	0.63	F = 5	1.45	1.13	0.030	16.56						
Median	9.0		36.76	10.37	0.288	120.19						

*M* male, *F* female, *BDNF* brain-derived neurotrophic factor, *IGF-1* insulin-like growth factor 1, *CBZ* carbamazepine, *R* risperidone, *MTPHE* methylphenidate, *HLP* haloperidol, *VPA* valproic acid, *No Tx* no treatment, *CGI-S* Clinical Global Impression-Scale, *ABC-T* Autism Behavior Checklist total, *ADI-T* Autism Diagnostic Interview total, *ATEC-T* Autism Treatment Evaluation Checklist total, *CARS* Childhood Autism Rating Scale, ^a,b,c^outliers detected by Grubb’s test, ^a^in autism group, n = 22, ^b^in medicated ASD, n = 14, ^c^in unmedicated ASD, n = 8.

**Table 2:** Characteristics of control subjects.

ID#	Age years	Sex	BDNF ng/ml	ProBDNF ng/ml	IGF1 ng/ml
C1	5	F	34.74	8.70	103.39
C2	5	M	38.07	14.56	71.43
C3	5	M	40.37	13.82	106.83
C4	5	M	43.35	10.94	84.8
C5	5	M	47.36	12.37	96.39
C6	6	M	7.14	13.94	65.71
C8	6	M	43.33	13.43	157.61
C11	6	M	42.96	11.50	95.24
C12	7	M	45.47	10.65	124.53
C13	7	F	38.1	8.23	108.7
C14	7	F	42.27	17.48	123.03
C15	7	M	27.95	17.48	113.51
C16	8	M	9.38	17.01	58.53
C17	9	M	35.56	14.90	89.44
C18	9	M	35.37	15.52	85.62
C19	9	F	34.04	13.95	107.17
C20	9	M	41.17	30.56^§^	127.78
C21	10	M	30.73	14.55	103.2
C22	10	M	34.63	17.32	53.63
C23	11	M	38.05	15.22	272.04
C24	11	M	26.56	19.85	47.52
C25	11	M	20.58	nd	46.63
C26	11	F	27.01	19.36	50.32
C27	11	F	21.35	16.28	230.54
C28	11	F	36.47	17.01	115.41
C29	12	M	25.1	16.53	141.43
C30	13	M	26.41	17.40	144.11
C32	14	F	43.08	11.09	100.22
C33	14	M	35.5	16.31	250.52
Mean	8.68	M = 21	33.99	15.21	115.31
SEM ±	0.54	F = 8	1.87	0.80	10.53
Median	9.00		35.53	15.06	105.11

*M* male, *F* female, *BDNF* brain-derived neurotrophic factor, *IGF-1* insulin-like growth factor 1, ^§^outlier detected by Grubb’s test, *nd* not determined.

The diagnosis of autism followed DSM-V criteria^1^. Additionally, subjects were evaluated according to the Clinical Global Impression (CGI-S) Autism Treatment Evaluation Checklist (ATEC), the Autism Diagnostic Interview-Revised (ADI-R), the Autism Behavior Checklist (ABC) and the Childhood Autism Rating Scale (CARS). Autistic children with a history of seizure or psychotic disorders, severe head injury, or any other acute or chronic mental disease, inflammatory illness, or comorbidities were excluded. Control participants with a history of any immunological or infectious illness, metabolic, psychiatric, or neurological disorders were also excluded. All recruited subjects were of normal weight and free of any active treatment with immunomodulatory drugs. All subjects had IQ scores within the normal range. Fifteen of the subjects with autism (Table 1, subjects A1–A15) were included in previously published studies^38,39^.

### Sample collection

Fasting peripheral blood samples were collected in the morning in 2 ml Vacutainer blood collection tubes and kept for 30 min at room temperature until the retraction of the clot. The blood was centrifuged for 10 min at 1000×*g* in a swinging bucket centrifuge pre-chilled to 4 °C. The serum was harvested, divided into aliquots, and stored at − 80 °C until the analysis. All experimental protocols were in accordance with the norms of the Advisory Scientific Committee and the Ethics Approval Board of the International Center for Neurological Restoration (CIREN).

### Neurotrophic factor analysis

BDNF, proBDNF and IGF-1 ELISA: Serum BDNF, proBDNF and IGF-1 were measured^40^ using the Human BDNF DuoSet ELISA kit DY248, Human proBDNF DuoSet ELISA kit DY3175 and Human IGF-1 Quantikine ELISA kit DG100 (R&D Systems, Minneapolis, MN, US). The ancillary kit was used for BDNF and proBDNF. For BDNF measurements, serum samples were diluted 75 ×; the results are shown as the mean of four readings for each sample, each reading representing an independent dilution. For proBDNF measurements, serum samples were diluted 20 × and represent the mean of two independent plates. For IGF-1 quantification, serum samples were pre-treated according to the manufacturer’s instructions (R&D Systems); final dilution of the samples was 100 ×. Samples and standards for IGF-1 ELISA were analyzed in duplicate, and the final values were the average of two readings, each reading representing a separate dilution. Absorbances for BDNF, proBDNF and IGF-1 were read at 450 nm, with a reference reading at 540 nm, using a Multiskan GO plate reader and SKANIT 3.2 software (Thermo-Fisher Scientific, Waltham, MA, USA). BDNF, proBDNF and IGF-1 serum levels were expressed in ng/ml.

### Data analysis

Statistical analyses were performed using SPSS 26 software (SPSS, Chicago, IL, USA) and GraphPad Prism software version 8.0.2 (GraphPad, La Jolla, CA, USA). The distribution of the data was examined by the Shapiro–Wilk normality test, and the presence of outliers was determined using the Grubbs' test. The significance of differences between groups was determined by 2-tailed unpaired *t*-test, Independent-Samples Mann–Whitney *U* test, Kruskal–Wallis test or 2-way ANOVA, according to the experimental design. Spearman’s correlation test was used to evaluate associations between variables. Statistical significance was set at p < 0.05.

### Ethical standards

The Ethical Committee of the International Center approved the experimental protocols as part of the National Program of Neuroscience and Neurotechnology (Code PN305LH013-056) in accordance with the Helsinki norms and rights for human research. Informed consent from parents or caregivers was obtained for the study. All methods were carried out in accordance with relevant guidelines and regulations.

## Results

### Subject characteristics

Tables 1 and 2 show the demographic data and serum measurements for subjects with autism and controls. BDNF and IGF-1 measures from many of the subjects with autism were previously published as part of an analysis of non-invasive brain stimulation effectiveness in these patients^39^.

The Shapiro–Wilk normality test revealed that no variables except age, CARS scores and BDNF were normally distributed within the autism group (n = 22). The Shapiro–Wilk normality test on medicated subjects (n = 14) and unmedicated subjects (n = 8) revealed normal distribution for BDNF and proBDNF and the ratio of proBDNF/total BDNF. IGF-1 levels in medicated subjects only were not normally distributed. No other variables passed the Shapiro–Wilk normality test. Grubbs' test detected one outlier in the autism group for proBDNF levels and ABC-T, ADI-T and ATEC-T scores. In the control group, one outlier for proBDNF levels and two outliers for the ratio of proBDNF/total BDNF were detected (outliers marked in Tables 1 and 2). In the medicated ASD group, one outlier was detected for proBDNF levels and in the unmedicated ASD group, one outlier was detected for IGF-1 levels. The outliers were included in all analyses. For some analyses we also presented values when outliers were removed.

A two-way Univariate Analysis of Variance, group × sex, showed no significant effect of group or sex and no group × sex interaction for IGF-1, BDNF or proBDNF levels (IGF-1 group p = 0.133, sex p = 0.789, group × sex p = 0.589; total BDNF group p = 0.829, sex p = 0.326, group × sex p = 0.133; proBDNF group p = 0.195, sex p = 0.899, group × sex p = 0.351. Therefore, subsequent analyses combined male and female subjects.

### Increased levels of serum IGF-1, no difference in serum BDNF and decreased serum proBDNF levels in subjects with autism compared to controls

**Figure 1:**
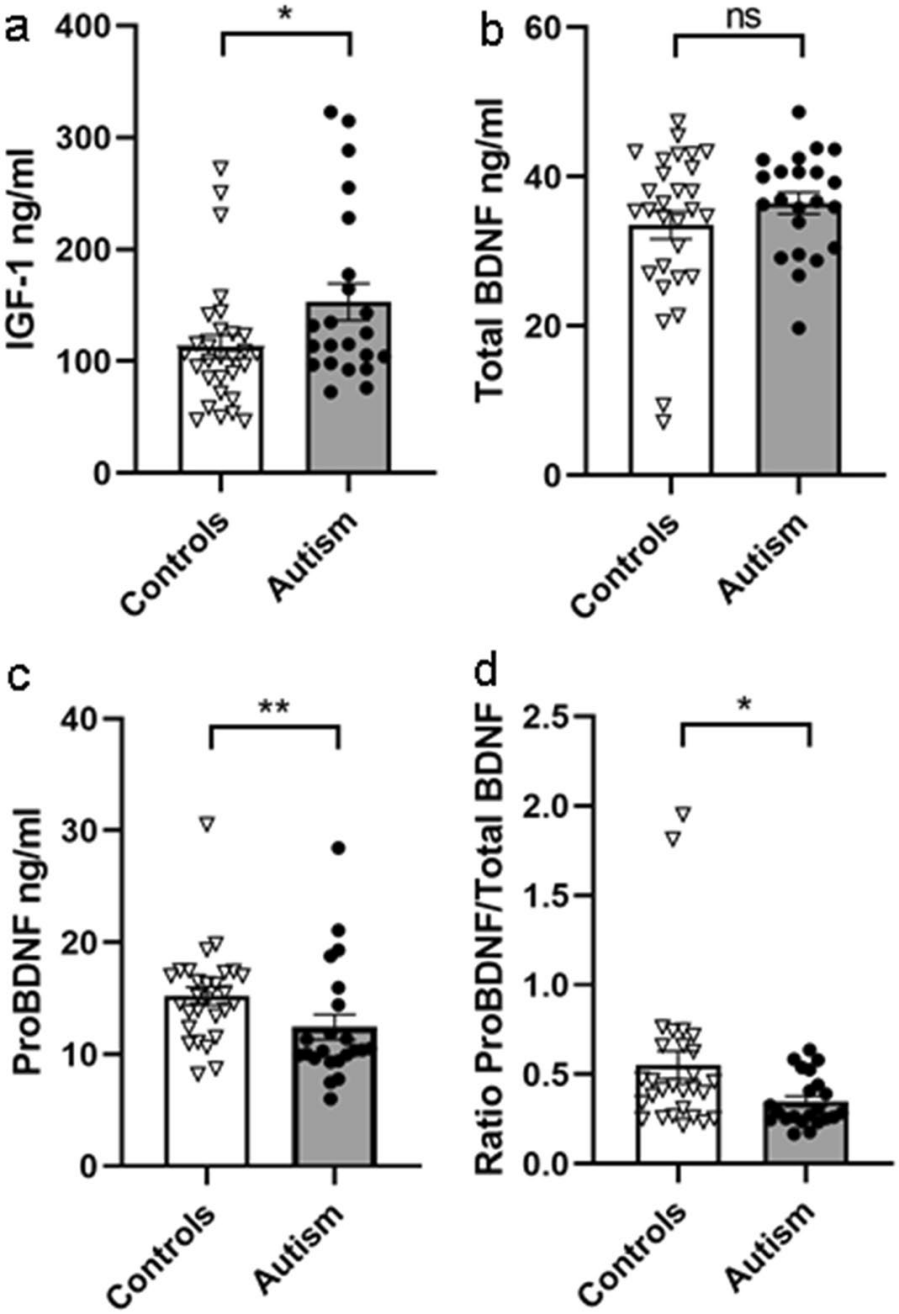
Serum protein levels measured by ELISA. (**a**) IGF-1, (**b**) total BDNF, (**c**) proBDNF and (**d**) the ratio of proBDNF/total BDNF immunoreactive protein levels in serum of controls (n = 29, or n = 28 where indicated) and subjects with autism (n = 22). 2-tailed Mann–Whitney test, IGF-1 *p = 0.037, total BDNF p = 0.350, *ns* not significant; proBDNF **p = 0.005, controls n = 28; proBDNF/total BDNF *p = 0.024, controls n = 28. Data are shown as mean ± SEM.

Mean IGF-1 immunoreactivity was significantly increased by 36% in the serum of subjects with autism compared to controls (Fig. 1a, 2-tailed Mann–Whitney test, p = 0.037, n = 51). The higher points in Fig. 1a represent older subjects. Serum BDNF immunoreactivity was not significantly different between the autistic group and controls (Fig. 1b, 2-tailed Mann–Whitney test, p = 0.350, n = 51). Serum levels of proBDNF were significantly lower in subjects with autism compared to controls (Fig. 1c, 2-tailed Mann–Whitney *U* test, p = 0.005, n = 50). This was also true after removing outliers A17 and C20 (2-tailed Mann–Whitney *U* test, p = 0.002, n = 48). The ratio of proBDNF to total BDNF was also reduced in the ASD group (Fig. 1d, 2-tailed Mann–Whitney *U* test, p = 0.024, n = 50). After removing two outliers from the control group, C6 and C16, there was only a strong trend towards significance (2-tailed Mann–Whitney *U* test, p = 0.055, n = 48).

There was no correlation between total BDNF and IGF-1, proBDNF and IGF-1 or between proBDNF and total BDNF serum levels (Spearman’s correlation BDNF vs. IGF-1 r_s_ = 0.236, p = 0.096, n = 51; proBDNF vs. IGF-1: r_s_ = − 0.245, p = 0.087, n = 50; proBDNF vs. total BDNF: r_s_ = − 0.176, p = 0.221, n = 50). However, there was a significant negative correlation between the ratio of proBDNF/total BDNF and IGF-1 levels when all samples were analyzed (Spearman’s correlation r_s_ = − 0.284, p = 0.046, n = 50). This was maintained in the autism group when analyzed separately (Spearman’s correlation ASD: r_s_ = − 0.468, p = 0.028, n = 22), but not in the control group (Spearman’s correlation controls: r_s_ = − 0.121, p = 0.540, n = 28).

### Different levels of serum proBDNF, but not BDNF or IGF-1, in medicated versus unmedicated subjects with autism

**Figure 2:**
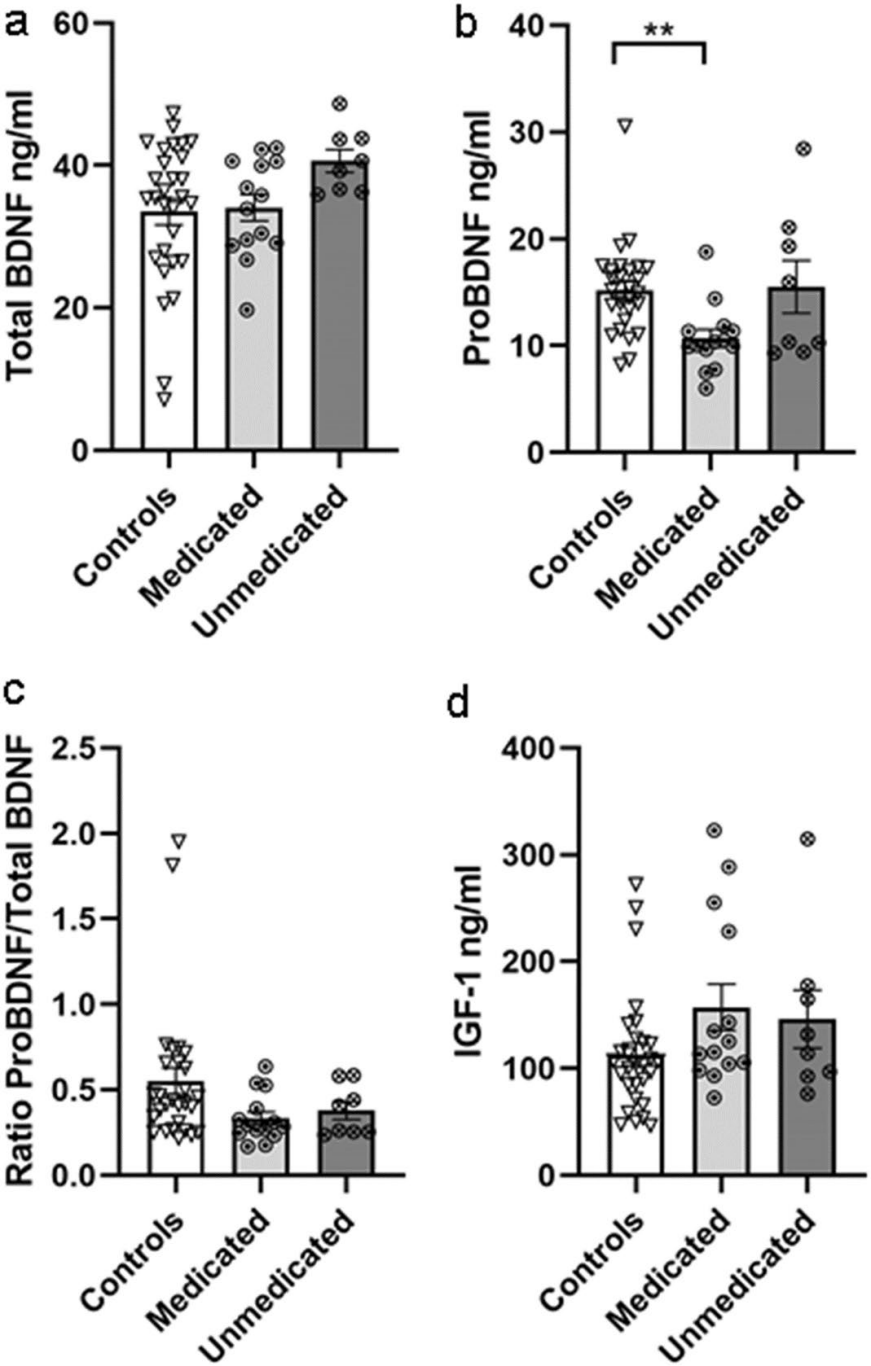
Serum BDNF and IGF-1 protein levels in unmedicated and medicated subjects. BDNF and IGF-1 immunoreactive protein levels in serum of unmedicated (n = 8) and medicated (n = 14) subjects with autism and in non-autistic controls (n = 29 or n = 28 where indicated). Kruskal–Wallis test, (**a**) total BDNF, p = 0.073; (**b**) proBDNF **p = 0.004 followed by Dunn’s test unmedicated vs. medicated p = 0.187, unmedicated vs. controls p > 0.999, medicated vs. controls **p = 0.003, controls n = 28; (**c**) proBDNF/total BDNF p = 0.070, controls n = 28; (**d**) IGF-1 p = 0.108. Data are shown as mean ± SEM.

There was a trend towards increased levels of serum total BDNF immunoreactivity in unmedicated subjects with ASD compared to subjects with ASD taking medication and to non-autistic controls (Fig. 2a, Kruskal–Wallis test, p = 0.073, Dunn’s test unmedicated vs. medicated p = 0.122, unmedicated vs. controls p = 0.096; medicated vs. controls p > 0.999).

In contrast, while serum proBDNF levels did not differ between unmedicated ASD and non-autistic control subjects, the levels of serum proBDNF immunoreactivity in medicated subjects with ASD were significantly lower than those in non-autistic controls (Fig. 2b, Kruskal–Wallis test, p = 0.004; Dunn’s test medicated vs. controls p = 0.003; unmedicated vs. medicated p = 0.187, unmedicated vs. controls p > 0.999). Removing outliers A7 and C20 from the analysis did not alter this result (Kruskal–Wallis test, p = 0.001; Dunn’s test medicated vs. controls p < 0.001; unmedicated vs. medicated p = 0.081, unmedicated vs. controls p > 0.999). There was a trend toward significant differences between groups in the ratio of proBDNF to total BDNF. This trend was lost after removing outliers C6 and C16 from the control group (Fig. 2c, Kruskal–Wallis test, p = 0.070, n = 50; p = 0.136, n = 48).

There were no significant differences between groups when serum levels of IGF-1 immunoreactivity were analyzed in ASD subgroups taking medication and unmedicated and controls (Fig. 2d, Kruskal–Wallis test, p = 0.108, n = 51).

### Correlations of IGF-1, BDNF and proBDNF serum levels with age

**Figure 3:**
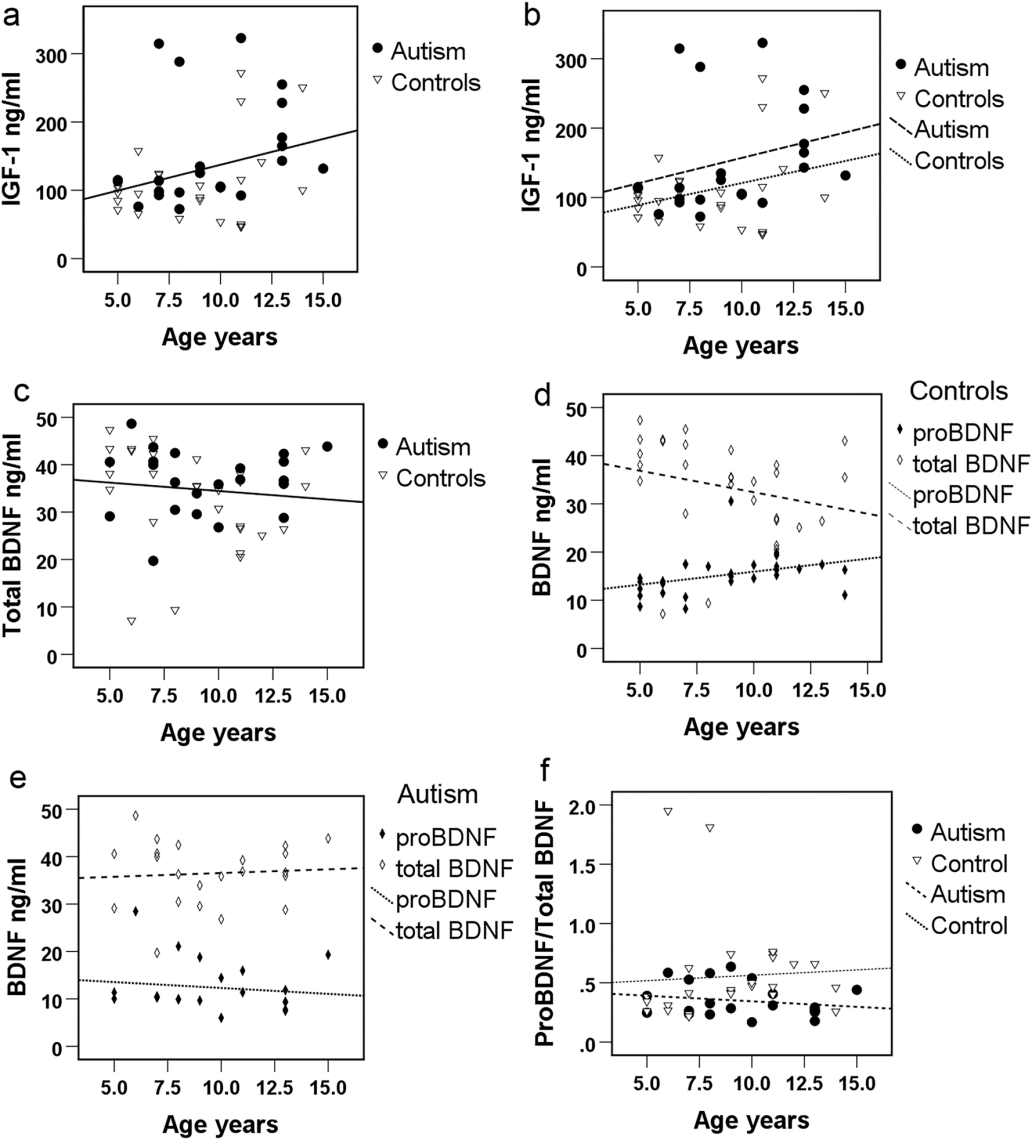
Correlations between serum IGF-1 and BDNF immunoreactivity and age. Spearman’s correlations: (**a**) IGF-1 correlation of all samples with age, r_s_ = 0.345, p = 0.013, n = 51. (**b**) IGF-1 correlation of each group separately with age (control group r_s_ = 0.207, p = 0.282, n = 29; autism group r_s_ = 0.429, p = 0.046, n = 22). (**c**) Total BDNF correlation of all samples with age, r_s_ = − 0.233, p = 0.101, n = 51. (**d**) Total BDNF and proBDNF correlations with age in the control group (total BDNF r_s_ = − 0.436, p = 0.018, n = 29; proBDNF r_s_ = 0.516, p = 0.005, n = 28). (**e**) Total BDNF and proBDNF correlations with age in the autism group (total BDNF r_s_ = − 0.010, p = 0.966, n = 22; proBDNF r_s_ = − 0.205, p = 0.359, n = 22. (**f**) Correlation of proBDNF/total BDNF in each group separately with age (control group r_s_ = 0.469, p = 0.012, n = 28; autism group r_s_ = − 0.075, p = 0.739, n = 22). Open triangles, controls; filled circles, autism; open diamonds, total BDNF; closed diamonds, proBDNF.

There was a significant positive association of serum IGF-1 immunoreactivity with age (Fig. 3a, Spearman’s correlation r_s_ = 0.345, p = 0.013, n = 51). Although our study did not reveal a statistically significant correlation between IGF-1 and age in the control group analyzed separately (Spearman’s correlation r_s_ = 0.207, p = 0.282, n = 29), the correlation with age was maintained in the autism group (Spearman’s correlation r_s_ = 0.429, p = 0.046, n = 22, Fig. 3b). Serum total BDNF immunoreactivity did not correlate with age (Fig. 3c, Spearman’s correlation r_s_ = − 0.233, p = 0.101, n = 51). When the groups were analyzed separately, in the control group (Fig. 3d), total BDNF was negatively correlated with age (r_s_ = − 0.436, p = 0.018, n = 29) while proBDNF was positively correlated with age (Spearman’s correlation r_s_ = 0.516, p = 0.005, n = 28). However, there was no correlation between total BDNF or proBDNF and age in the autism group (Fig. 3e, Spearman’s correlation BDNF r_s_ = − 0.010, p = 0.966; proBDNF r_s_ = − 0.205, p = 0.359, n = 22). There was a correlation in the control group between the ratio of proBDNF/BDNF and age (Fig. 3f, Spearman correlation r_s_ = 0.469, p = 0.012, n = 28), which became stronger after removal of outliers C6 and C16 (Spearman correlation r_s_ = 0.602, p = 0.001, n = 26), while there was no significant correlation between proBDNF/BDNF ratio and age in the ASD group (Spearman correlation r_s_ = − 0.075, p = 0.739, n = 22). The correlation of the ratio of proBDNF/total BDNF with age when all subjects were analyzed together was not significant either without or with outliers (Spearman correlation r_s_ = 0.247, p = 0.091, n = 48 and r_s_ = 0.247, p = 0.196, n = 50 respectively).

### Correlations of autism diagnostic scores with serum IGF-1 and BDNF levels

**Figure 4:**
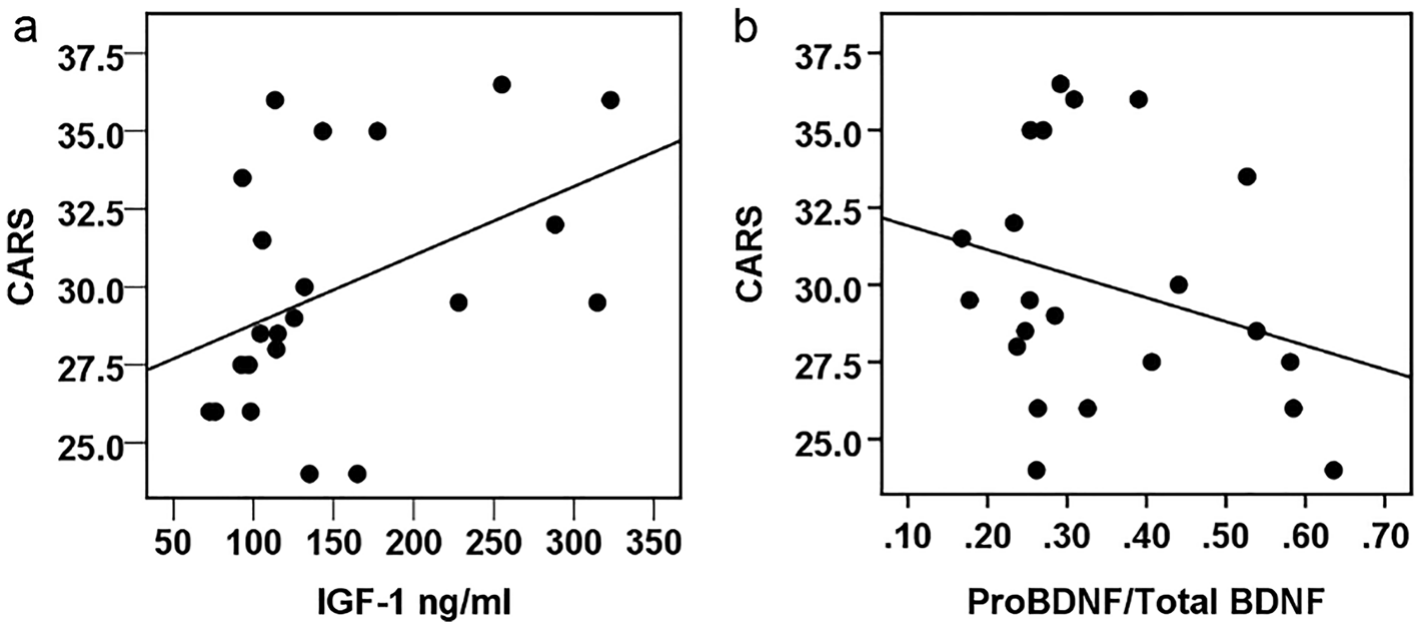
Correlations between Childhood Autism Rating Scale (CARS) scores and IGF-1 and proBDNF/BDNF. (**a**) IGF-1 immunoreactivity, Spearman’s correlation r_s_ = 0.486, p = 0.022 and (**b**) ratio proBDNF/BDNF, Spearman’s correlation r_s_ = − 0.407, p = 0.060. n = 22.

There was a significant positive correlation between Childhood Autism Rating Scale (CARS) scores and IGF-1 immunoreactive levels (Fig. 4, Spearman’s correlation r_s_ = 0.486, p = 0.022, n = 22). There was no correlation of CARS scores with total BDNF or with proBDNF immunoreactivity levels (Spearman’s correlation r_s_ = − 0.069, p = 0.760 and r_s_ = − 0.238, p = 0.287, n = 22, respectively), however, there was a trend toward a negative correlation between the proBDNF/BDNF ratio and CARS scores (Spearman’s correlation r_s_ = − 0.407, p = 0.060, n = 22). We did not detect any relationship between CGI-S, ATEC, ADI-R or ABC scores with IGF-1, BDNF or proBDNF immunoreactivity.

## Discussion

Growth factors are a set of proteins that play a crucial role in cellular growth, proliferation, and differentiation in the CNS. Because of their role in CNS development, growth factors have been studied in neuroplasticity modulation and behavioral effects, with a particular emphasis on neurodevelopmental disorders^6,11,41,42^. Insulin-like growth factors (IGFs) are a family of proteins forming part of a complex signaling pathway including IGFs (i.e., IGF-1 and IGF-2), IGF receptors, binding proteins, and proteases. IGF-1 is neuroprotective and is involved in brain development^44–45^. Changes in IGF-1 signaling are implicated in the pathogenesis of neurological and psychiatric disorders including autism and major depressive disorder^6,46,47^. Both BDNF and IGF-1 are known to increase signaling through the PI3K-Akt-mTOR pathway^26,48^, a signaling pathway that is decreased in idiopathic ASD^5,49–52^. Because of its pharmacological properties, IGF-1 has been suggested as a treatment for autism^53,54^. In fact, IGF-1 has been useful in Rett syndrome (RTT) and in Phelan–McDermid Syndrome, both neurodevelopmental ASDs^10,54–55^. Trofinetide, an analog of the amino-terminal tripeptide of IGF-1, has also induced clinically relevant improvements in RTT^56^. IGF-1 may exert its effects in RTT by acting on the mTOR pathway, which is down-regulated in RTT^57^. Several clinical trials are currently testing the safety and efficacy of IGF-1 or IGF-1 analogues in RTT patients, and preliminary analysis demonstrates improvement in cardiorespiratory parameters, disease severity, and some behavioral and EEG measures of mood and anxiety^10,53,54,56^. Despite the promise of IGF-1 treatment for autism, CNS IGF-1 levels do not differ between subjects with autism and controls. In CSF of children with autism younger than 5 years old, IGF-1 levels are reportedly lower than in controls^28,30^. However, IGF-1 levels in CSF from children with autism at 5–15 years old were not different from controls at those ages^30^, consistent with a lack of difference in IGF-1 levels in post-mortem brain tissue^29,33^.

In contrast, several reports, including this one, document changes in IGF-1 levels in the periphery of children with autism^30–32^. The significantly increased peripheral blood levels of IGF-1 immunoreactivity observed in our subjects with autism compared to controls are in agreement with the results reported by Mills et al.^32^ and Şimşek et al.^34^, who showed IGF-1 increases in ASD plasma and serum, respectively. Mills et al.^32^ restricted their study sample to only boys between 4 and 8 years old, whereas Şimşek et al.^34^ found a significantly higher level of IGF-1 protein in a mixed population of boys and girls ages 4–12, consistent with the broader age range of 5–15 years and both sexes (25% females) in our study. Although IGF-1 may be a beneficial treatment for ASD, there appears to be no alteration in IGF-1 levels in autism CNS tissue or fluids compared to controls^29,30,33^. Increased blood levels of IGF-1 reported here, coupled with decreased levels of IGF-1 in urine of young subjects with autism^31^, instead suggest reduced excretion of peripheral IGF-1 in autism subjects compared to controls. Reduced excretion might be due to increased binding of IGF-1 by the IGF-1 binding protein, insulin-like growth factor binding protein 3 (IGFBP-3) that is increased in blood from autism patients^32^. Analysis of serum IGF-1 in children younger than 4–5 years old and of urine IGF-1 in older children would be of interest to clarify if reduced IGF-1 excretion is responsible for increased serum IGF-1 levels in autism.

In agreement with previous reports^58–61^, we found that serum IGF-1 immunoreactivity increases with prepubertal age. The IGF-1 levels of all measured samples were significantly correlated with the age of the subjects. Although our study did not show a statistically significant correlation between IGF-1 immunoreactivity and age in the control group analyzed separately (which may be attributable to our small sample size), the statistically significant correlation in the autism group and the parallel fitting lines in Fig. 3b suggest a relationship in each group. The parallel fitting lines demonstrate also, that across the tested range of 5–15 years of age, serum IGF-1 levels of children with autism were higher than levels of controls.

In this study, we found a significant positive correlation between Childhood Autism Rating Scale (CARS) scores and IGF-1 immunoreactivity. Why IGF-1 should correlate with CARS scores but not with other measures of autistic behavior is unclear.

Among the different families of growth factors, the neurotrophin family has received much attention for its role in neuropsychiatric conditions such as neurodegenerative disorders (e.g., Alzheimer’s disease), neurodevelopmental disorders (e.g., autism), and psychiatric disorders (e.g., depression, bipolar disorder)^5,8,42,62–64^. BDNF, in particular, is a neurotrophin that is abundantly expressed in the adult CNS, playing a prominent role in cognitive processes such as learning, memory, and behavioral consolidation^16,65^. BDNF and its precursor, proBDNF, are also widely expressed in the developing and postnatal brain. BDNF and proBDNF exhibit opposite activities on synapses, with BDNF signaling spine maturation, neurite outgrowth, survival and LTP while proBDNF signals spine weakening, neurite retraction, apoptosis and LTD^20,21^. BDNF and proBDNF regulate synaptogenesis and neuronal migration as well as influence synaptic plasticity and cortical circuitry development, all relevant for autism^12,13,66^.

In this study, we did not find statistically significant differences in serum BDNF-immunoreactive levels between subjects with autism and controls. However, proBDNF levels were lower in serum of subjects with ASD compared to controls. These results are in contrast to Bryn et al.^67^, who found increased BDNF, but not proBDNF levels, in plasma of children with ASD compared to controls. This is also inconsistent with our findings in post-mortem brain tissue, in which we found higher proBDNF levels in ASD fusiform gyrus than in controls^9^. These discrepancies may reflect poor efflux of proBDNF from brain compared to BDNF, differences in commercial proBDNF and BDNF ELISA assays and in Western blotting versus ELISA quantification of proBDNF, or altered proBDNF secretion from platelets into peripheral blood compared to BDNF^68^. Le Blanc et al.^68^ noted very different proBDNF/BDNF ratios in serum versus plasma, supporting the latter hypothesis.

When we stratified subjects with ASD according to medication/no medication, we found that subjects treated with mood stabilizers including carbamazepine, risperidone, haloperidol, methylphenidate and valproic acid exhibited lower serum proBDNF-immunoreactive levels than controls, suggesting that the inclusion of drug-treated subjects in the ASD group could be responsible for the reduced proBDNF levels when all ASD subjects were grouped together. Interestingly, neither BDNF nor IGF-1 serum levels differed between medicated and unmedicated subjects.

Previous findings support increased BDNF immunoreactivity in post-mortem ASD brain tissue compared to controls, in addition to decreases in the full-length isoform of the BDNF receptor, TrkB-FL, and its downstream PI3K-Akt-mTOR signaling pathway^8,9,22^. Likewise, in the periphery, most but not all groups report higher BDNF-immunoreactive levels in ASD compared to controls^24,25^. Western blots indicate that, in the brain, the increase in BDNF immunoreactivity in subjects with ASD is due to higher levels of proBDNF^9^. Since commercial BDNF ELISAs generally recognize both proBDNF and BDNF but with differing affinities, the contribution of proBDNF to changes in peripheral BDNF levels is difficult to discern without performing Western blots to distinguish BDNF isoforms. Nevertheless, in this study we used a commercial proBDNF ELISA to determine the effects of medication on the proBDNF isoform. Although the proBDNF ELISA failed to confirm higher amounts of serum proBDNF in subjects with ASD versus controls, normal or reduced levels of proBDNF may be the result of medications taken by the ASD group. Indeed, while proBDNF levels in drug naïve ASD subjects were comparable to control levels, proBDNF levels in medicated ASD subjects were significantly lower than in controls. This suggests that the normalization of BDNF levels by mood-stabilizing drugs in subjects with autism may include a reduction in proBDNF, which represents a portion of total BDNF levels. This possibility is supported by a report that tissue plasminogen activator (t-PA), an activator of plasmin that processes proBDNF to BDNF, is increased by valproate^69^. Unfortunately, previous reports of increased t-PA in children with autism^70^ do not mention the medication status of the children.

Current pharmacological treatment of ASDs includes antidepressants, anxiolytics and atypical antipsychotics^36,71–73^. Only two medications have been approved by the US FDA as useful for alleviating irritability, aggression and self-injury and stereotypy disorders, but not sociability defects, in autism: risperidone (Risperdal^®^), and aripiprazole (Abilify^®^)^74,75^. Normalization of BDNF levels following effective treatment with these antipsychotics has been reported^76,77^. Serum proBDNF levels following these treatments have not been investigated.

Methylphenidate (MTPHE) is the most commonly used psychostimulant in children with attention deficit hyperactivity disorder, as it significantly reduces hyperactivity and improves social skills in these children^78,79^. MTPHE affects predominantly dopamine and noradrenergic systems, with evidence of its role in neuroplasticity and behavior via dopamine D3 receptors and BDNF^80,81^. Although the literature is mixed, two reports demonstrate reductions of BDNF in ADHD children treated with MTPHE^82,83^ and two reports demonstrate increases^84,85^. ProBDNF levels have not been investigated in MTPHE-treated subjects with autism.

Carbamazepine (CBZ) and valproate (VPA) are two of the most common therapeutic drugs used to stabilize mood disorders and are also used frequently in autism. Both increase BDNF mRNA expression in individuals with temporal lobe epilepsy^86^. VPA is a histone deacetylase inhibitor that is known to increase BDNF expression in hippocampus, cortex and amygdala when given to adult animals^87^. However, most of these studies have assayed BDNF at the mRNA rather than the protein level, and none has attempted to distinguish BDNF from proBDNF. Counter-intuitively, the one subject in our study treated with VPA exhibited the lowest BDNF levels of any of the autism subjects but did not exhibit unusual proBDNF levels. Although proBDNF, not BDNF, levels are elevated in post-mortem brain tissue of humans with autism^9^, proBDNF levels have not been assayed following VPA administration in humans with autism.

Our report is one of the first to examine both BDNF and proBDNF in peripheral blood of autistic children. Another study^67^ assayed plasma rather than serum BDNF and proBDNF in a slightly older study population (average age 11 instead of our 9 years) and did not test the correlation of BDNF or proBDNF with age. We report here that serum levels of both BDNF and proBDNF were associated with age, but only in the control group. BDNF levels decreased with age, whereas proBDNF increased with increasing age. This suggests that processing of proBDNF to mature BDNF becomes progressively less efficient over time such that proBDNF represents a greater proportion of the total BDNF immunoreactivity in older subjects compared to younger. This is consistent with reports that both BDNF and proBDNF are crucial for synaptic remodeling during development^14,88^, and that BDNF gives way to proBDNF as circuits depend less on growth and more on fine tuning. The lack of correlation between these BDNF isoforms and age in the autism group provides further evidence that disruption in BDNF isoform balance during development is associated with defects in the establishment and maintenance of functional neuronal networks^9^.

A major limitation of this analysis is the small group size and high variability in the unmedicated ASD group. Further experiments are required to verify the isoform of BDNF that is elevated in serum. Notably, BDNF serum levels are affected by the Val66Met BDNF polymorphism^89,90^. The genotype of the study subjects is not known, and thus the degree to which the Val66Met BDNF polymorphism might have increased variability is unknown.

In summary, we found a significant increase in serum IGF-1 and a significant decrease in serum proBDNF in young boys and girls with mild to moderate autism compared to controls. The increase in serum IGF-1 levels may be due to decreased excretion in urine. IGF-1 levels were positively correlated with age and did not vary with medication status. Total BDNF levels in subjects with autism were similar to controls, regardless of medication status. However, proBDNF levels in subjects with autism, in particular, those being treated with medication, were lower than in controls, suggesting that the beneficial actions of these medications may include lowering proBDNF levels. The significant elevation in IGF-1 in the autism group and the reduced proBDNF immunoreactivity in medicated subjects with autism compared to controls reinforces the involvement of those two growth factors in the pathophysiology and treatment of autism. The role of neurotrophic factors in autism should be studied further, with necessary inclusion of both sexes, larger study populations (particularly medication-naïve subjects) and, in the case of IGF-1 serum levels, inclusion of subjects younger than 4 years old.

## Data Availability

All data generated or analysed during this study are included in this published article.
